# Supplementary material for: An Aptamer That Rapidly Internalizes into Cancer Cells Utilizes the Transferrin Receptor Pathway
Source: Cancers (Basel). 2023 Apr 14;15(8):2301. doi: 10.3390/cancers15082301 (PMC10136559; doi:10.3390/cancers15082301)
Supplement: Supplementary file 1 [file cancers-15-02301-s001.zip › cancers-2287485-supplementary.pdf]

An Aptamer that Rapidly Internalizes into Cancer Cells Utilizes the Transferrin Receptor Pathway

Xirui Song [1,2], Haixiang Yu [1], Cynthia Sullenger [3], Bethany Powell Gray [1,4], Amy Yan [1], Linsley Kelly [1] and Bruce Sullenger [1,2,*]

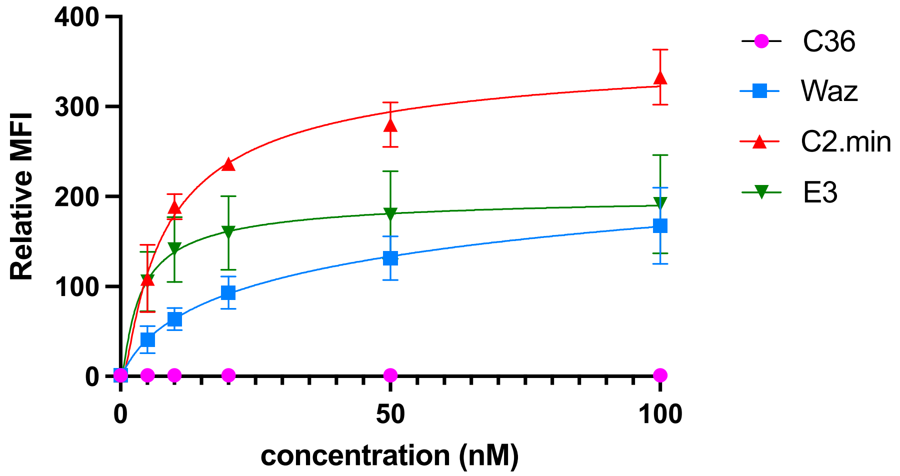

Figure S1. DL650-labeled aptamer binding signals on immobilized human TfR 1 protein. A wider concentration range of DL650-aptamers was used (0-100 nM). The apparent K_D of Waz and B_{max} of three aptamers in Table 1 is generated based on this figure.

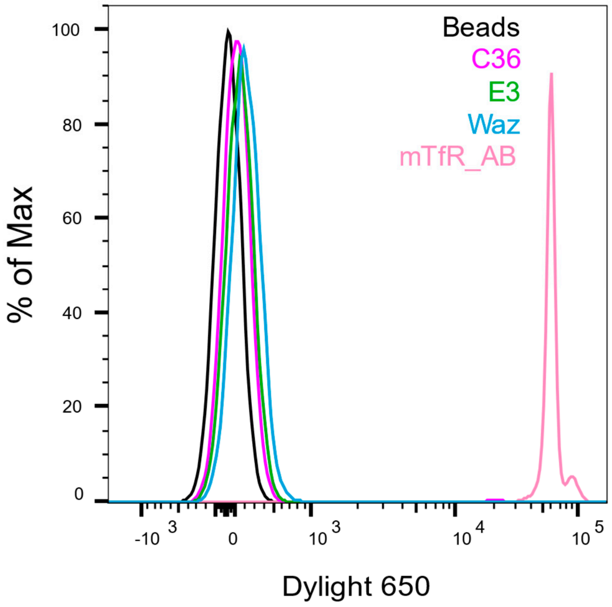

Figure S2. C36, E3, and Waz do not bind to mTfR protein. DL650-labeled C36, E3, Waz, and mTfR antibodies were added to the immobilized mouse transferrin receptor. The black line was the signal when no aptamer/antibody was

added to the protein. C36, E3, and Waz are shown in pink, green, and light blue, respectively. The antibody signal is shown in light pink.

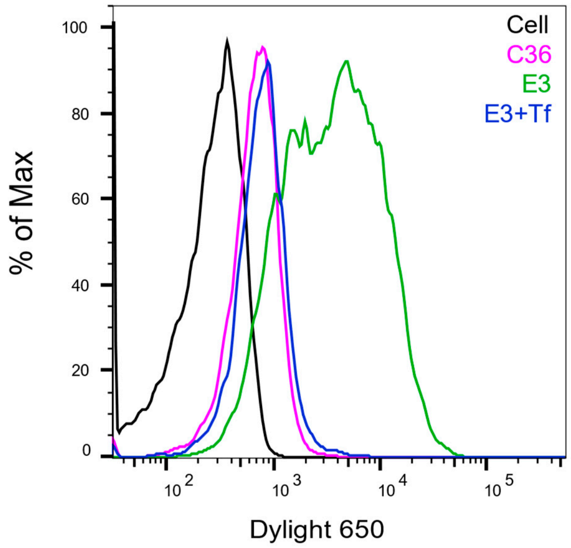

Figure S3. The binding signal of DL650-E3 on B16-hTfR cells can be competed off by adding human holo-Tf. The black line was the signal when no aptamer was added to the B16-hTfR cells. Signals of DL650-C36 and DL650-E3 independent of human Tf were shown in pink and green, respectively. When B16-hTfR cells were incubated with 1mg/ml of human holo-Tf, the signal of DL650-E3 was presented here in dark blue.

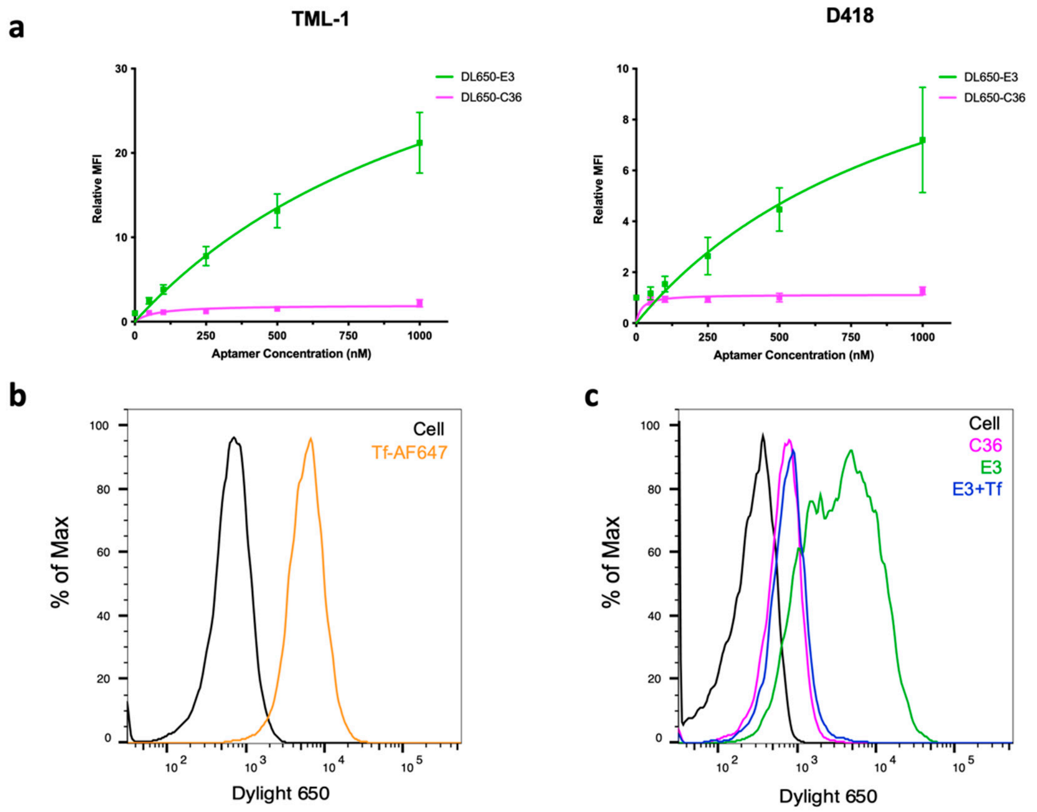

Figure S4. E3 binds D418 canine cancer cells, and it is in a TfR-dependent pathway. (a) binding curves of DL650-E3 and DL650-C36 on two canine cancer cell lines, TML-1 and D418. (b,c) The black lines were the signal when no dye-labeled molecules were added to the D418 cells. Signals of (b) AF647-labeled human transferrin (Tf-AF647), (c) DL650-C36, and DL650-E3 were shown in orange, pink, and green, respectively. Signals of DL650-E3 on D418 pre-incubated with 1mg/ml human holo-Tf were shown in blue.

Supplemental Methods

Binding analysis on a wider range of aptamers

Dynabeads His-Tag Isolation & Pulldown beads (Invitrogen) were washed with SB1T buffer and then incubated with his-tagged human transferrin receptor 1 (Sino Biology) for 30 min at room temperature. Meanwhile, DL650-labeled aptamers were folded in SB1T buffer for 5 min at 65°C, followed by a 15 min slow cool to room temperature. After incubation, the bead-protein complexes were washed and split into separate tubes to get 2 pmol protein/tube. Aptamers were serially diluted by 2-fold and added into each tube with 1 mg/ml ssDNA blocked SB1T buffer. Samples were incubated at 37°C for another 30 min. Next, proteins were collected via a magnetic field and washed with SB1T. Fluorescence intensities were collected via CytoFlex flow cytometer (Beckman Coulter).

Aptamer/antibody Binding on mTfR

Dynabeads His-Tag Isolation & Pulldown beads (Invitrogen) were washed with SB1T buffer and then incubated with His-tagged mouse transferrin receptor (Sino Biology #50741-M07H) for 30 min at room temperature. DL650-C36 and DL650-E3 were folded in SB1T buffer for 5 min at 65°C, followed by a 15 min slow cool to room temperature. After incubation, the bead-protein complexes were washed and split into separate tubes to get 2 pmol protein/tube. 1 mg/ml ssDNA was added into the aptamer solution where the final concentration of aptamer was 200 nM in 50 µl solution. APC-mTfR antibody (Invitrogen, clone R17217) and its isotype control were used in SB1T buffer with 1%

BSA. Samples were incubated at 37°C for another 30 min. Next, proteins were collected via a magnetic field and washed with SB1T. Fluorescence intensity was collected via CytoFlex flow cytometer (Beckman Coulter).

Binding Curves and Competition assay on D418 and TLM-1 cells

For cell-based binding curves, TLM-1 and D418 cells were seeded in a 24-well flat bottom tissue culture plate at 100,000 cells/well. After a 48 hr incubation at 37°C, 5% CO_2, and 95% relative humidity, old media was replaced by 180 µl 1.11 mg/ml ssDNA in complete media. The plate was returned to the incubator for 1 hr. Meanwhile, DL650-E3 and DL650-C36 were folded in DPBS+/+ (DPBS with Ca^{2+} and Mg^{2+}; Thermofisher) at a concentration of 10 µM. RNA solutions were heated at 65°C for 5 min and cooled down on the ice for another 5 min. The RNA solutions were serially diluted to prepare them at different concentrations. 20 µl RNA solution was added to each well, followed by 1 hr incubation. The cells were then washed twice with PBS, trypsinized off from the plate, quenched with complete media, and collected via centrifuge at 350xg for 5 min. After washing twice with PBS, cells were resuspended in 150 µl flow buffer (PBS+1% BSA) containing 1 µg/ml 7-aminoactinomycin (7-AAD) live/dead staining and incubated at 4 °C for 15 min. The fluorescent signals were collected on a CytoFlex flow cytometer (Beckman Coulter). Binding curves were generated using GraphPad Prism.

30,000 D418 cells were plated in a 96-well plate a day before. As described in other cell-based binding assays, 90 µl 1.1 mg/ml ssDNA in complete media was added into each well. For cells pre-incubated with human transferrin, each well received 1.1 mg/ml Tf in 1.1 mg/ml ssDNA-blocked complete media. After a 1 hr incubation, refolded DL650-E3, DL650-C36, and Tf-AF647 (Thermofisher #T23366) were added to the designated well at a final concentration of 500 nM. The collection and analysis steps were the same as described in the "Binding Curves on Recombinant Protein and Canine Cancer Cells".
